# Quality assurance of the university medical education, hospital services and traditional pharmaceutical products of the Bhutanese *So-wa-rig-pa* health care system

**DOI:** 10.1186/s12906-016-1270-2

**Published:** 2016-08-12

**Authors:** Phurpa Wangchuk, ᅟ Tashi

**Affiliations:** 1 Centre for Biodiscovery and Molecular Development of Therapeutics, Australian Institute of Tropical Health and Medicine, James Cook University, Cairns Campus, Townsville, QLD 4870 Australia; 2 Menjong Sorig Pharmaceuticals, Ministry of Health, Thimphu, Bhutan

**Keywords:** Quality assurance system, Quality control parameter, Bhutanese *So-wa-rig-pa* medicine, Integrated health care services, Traditional medical education, Traditional medicine hospital, Menjong sorig pharmaceuticals

## Abstract

**Background:**

The Bhutanese *So-wa-rig-pa* medicine (BSM) was integrated with the allopathic (modern) health care system in 1967. Ever since the health care integration policy was implemented, the BSM has gone through many phases of quality improvement and changes including the establishment of one university-based institute, 58 hospitals and Basic Health Units (BHU)-based health care services, and one traditional medicine factory. The BSM provides primary health care services to more than 20–30 % of patients who visit hospitals and BHU on a daily basis. However, there has been no study covering the quality assurance system of BSM. Our paper addresses this information gap.

**Methods:**

This study was an observational ethnographic study supported by phenomenological understanding and content analysis of the data. The information was triangulated through consultation with the BSM practitioners (discussion (*N* = 8)) and personalized in-depth question-answer sessions using electronic protocols (*N* = 5). These participants comprised BSM educationists, clinical physicians, researchers, production and the quality assurance staff who were selected using convenience and purposive sampling method. The relevant *So-wa-rig-pa* information and literature were obtained from the government policy documents, official websites, scientific papers and the traditional medical texts. This study is enhanced by our practical observations and first-hand experience with BSM while working as the researchers at the Ministry of Health in Bhutan. In addition, the information in this paper is crosschecked and authenticated by five *So-wa-rig-pa* practitioners of Bhutan.

**Results:**

The study highlights the following: a) The BSM receives both the government and people’s support, b) The quality assurance system have been developed by integrating the traditional empirical knowledge and modern scientific protocols, c) There exist three administrative and functional organizations responsible for providing the quality BSM health care services in Bhutan, d) Extensive standard treatment guidelines and Quality documentation system exist for BSM as required by the regulatory bodies in Bhutan. The paper also recommends appropriate future directions for BSM.

**Conclusions:**

The BSM plays significant role in the primary health care system of the country. Consequently, the quality, safety and efficacy of BSM has been given priority by the Bhutan government. Many scientific protocols were integrated with the traditional quality approaches and further scientific studies are still required to improve its quality.

## Background

The use of traditional medicine (TM) or complementary and alternative medicine (CAM) is widespread around the world. It is estimated that approximately 85–90 % of the world’s population including developed nations use traditional or alternative or herbal medicines [1]. Its use is increasing worldwide [2]. With the growing use of TM, demand has grown for evidence on the quality, safety and efficacy of TM products and practices. Even in Bhutan with a strong history of TM, few doctors with a scientific outlook express reservations or are skeptical about the purported benefits of TM. Therefore, scientific validation of TM and creation of standard research data or knowledge to address the issues of quality, safety and efficacy has become a pressing issue. Creation of such scientific knowledge can help create the basis for better health care integration and an evidence-based health system that is more respectful towards local practices [3] and foster better collaborations across medical professionals.

Realizing the importance of TM, the World Health Organization (WHO) exercised the following: a) a need for common understanding of what constitute TM, b) the integration of TM with allopathic medicine, and c) the measures to improve the quality, safety and efficacy of TM [4]. To facilitate the development of the quality control system, the WHO also developed general standard policies, strategies, frameworks, guidelines, protocols, standard operating procedures, regulations and bylaws [2, 4–7]. While the TM in many countries are struggling to meet these WHO quality requirements-mainly for the lack of government’s support, the Bhutanese traditional medicine (BTM) or the Bhutanese *g.so-ba-rig-pa* (pronounced as *So-wa-rig-pa*) medicine (BSM) has gained roots in the mainstream health care system of the country.

The BSM enjoyed wider people’s acceptance, received the successive Kings’ and the government support and was integrated with the allopathic or modern medicine in 1967. It started with a small dispensary in 1968 and developed into one of the complex organizations in the country with three main BSM management sectors: Department of Traditional Medicine Services (DTMS), Menjong Sorig Pharmaceuticals (MSP), and the Faculty of Traditional Medicine (FoTM). The DTMS under the Ministry of Health (MoH), looks after one National Traditional Medicine Hospital (NTMH), two regional referral traditional hospitals (Monger and Gaylegphu), 17 traditional medicine hospitals and additional 38 traditional medicine units established alongside major Basic Health Units (BHU) in the country. These units treat more than 20–30 % of the total daily out-patients visiting the hospitals and other health centers on a daily basis [8]. The MSP conducts research, quality assurance and quality control of raw materials and manufactured herbal products, medicinal plants collections, and processing of traditional medicines, which are distributed freely to the traditional medical centers across the country. The medicinal plants collection program facilitates the economically disadvantaged yak herders and other farmers to earn income and elevate their socio-economic status. It also helps in the conservation of pristine environment and has potential to facilitate the growth of herbal industry as well as the biodiscovery projects based on their ethnobotanical information. The FoTM under the Khesar Gyalpo University of Medical Science of Bhutan (KGUMSB) trains human resources required for catering the traditional medical services to the people.

All these three organizations are together responsible for the preservation of rich traditional medical culture and in providing quality, safe and effective traditional health care services. Ensuring the quality or ethnoquality of the BSM has direct implications on its long-term sustainability and the preservation of rich traditional medical knowledge. We have coined the term ethnoquality for the first time here and we defined it as “The cross-cultural study of the traditional knowledge and customs of how various ethnic healers/traditional medicine practitioners perceive and monitor the quality of medicines”. In their continuous efforts (more than 46 years) to improve the ethnoquality and preserve this age-old medical tradition, the FoTM, DTMS and MSP has undertaken various programs, measures and strategies. However, there is lack of proper studies or published information on what has been done so far to improve the quality of BSM. Not many people are aware of (including Bhutanese) whether there exists any proper policy and the quality management system while providing traditional *So-wa-rig-pa* medicine in the country.

This raises many questions. Is this medical system accepted by the people as a complementary health care system? Is it financially supported and protected by the government to be an integral part of Bhutanese health care system? Whether or not if there is any problem with the quality, safety and efficacy of the *So-wa-rig-pa* medicine? What are the quality assurance systems, practices and pillars that have been developed by the FoTM, NTMH and MSP? How is the quality of traditional *So-wa-rig-pa* medical education monitored and maintained by FoTM? How is the quality of traditional health care services managed by the traditional medicine hospital/units in the country? How is the quality and safety of medicines/drugs monitored and managed by MSP? Is there any scientific quality parameters and standards developed for assuring the quality, safety and efficacy of the medicines? Is there any scientific research carried out to make the BSM evidence-based medicine? What are the current quality deficiencies and how can they be addressed?

Having these research questions answered would give insights into the quality assurance system and the practices of an integrated health care services of Bhutan that has potential for providing the managerial lessons for other *So-wa-rig-pa* practicing countries and TM entrepreneurs including Tibetan medicine. Therefore, this study was conducted to determine what has been achieved in relation to improving the quality, safety and effectiveness of the traditional *So-wa-rig-pa* education, health care services and medicine production. It also sheds light on the existing government policies and the regulations related to the BSM and provide future directions.

## Methods

### Concepts and design

This study was derived from an on-going phenomenal changes involving Bhutanese *So-wa-rig-pa* medicine ever since its integration with the allopathic (western) medical system in 1967. The concept was to describe, explain and present to an international audience its quality assurance system and practices that were either modified in-situ based on traditional empirical knowledge or borrowed from western medicine quality control systems. Thus, our study design/framework includes ethnography, phenomenology and content analysis. In our ethnographic approach, we have associated ourselves as the researchers (both trained in western education system) for more than 13 years at the MSP and have worked closely with the Bhutanese *So-wa-rig-pa* communities - mostly traditional physicians at the FoTM, NTMH and MSP. These engagements with the practitioners helped us gain insight into their condition and perception of health system while also shaping our phenomenological understating including *So-wa-rig-pa* philosophy and historical events. Our own personal experience and first-hand observational work enabled us to compile and synthesise the existing information.

This observational data was then supported by the content analysis of the existing body of literature (both scientific and traditional) including traditional medical textbooks, health system policy documents, regulatory frameworks, traditional medicine education curriculum, disease classification and treatment guidelines, diagnostic methods and treatment practice records, quality assurance guidelines, standard manufacturing instructions, product profiles, medicinal plants collection reports and other documentation practices of three organizations (FoTM, NTMH, MSP) that were made available at the time of study. This information sources were extracted from the existing library, government websites, policy documents, acts, regulations, guideline books and the published scientific literature that are all retained in our research notes and listed in the bibliography. Some contents were obtained as previously described [8]. The search terms such as: ‘Bhutanese *So-wa-rig-pa* medicine’, ‘Bhutanese traditional medicine’, ‘Bhutanese medicinal plants’, ‘health policy’, ‘drug policy’, ‘medicine act’, ‘quality assurance’ and ‘quality control parameters’ were used for the content analysis involving the internet and relevant databases. For obtaining specific references and guidelines that are used for improving the quality of health care services, traditional medical education and the production of medicines, a standard data entry form was developed and distributed to relevant study participants representing FoTM, NTMH and MSP who were then requested to fill the form with the latest references used at these three organizations to guide the quality assurance practices. To clarify, crosscheck and authenticate the unstructured data that we have observed and recorded through ethnographic and content analysis methods, we have also consulted more than five *Drungtshos* (*So-wa-rig-pa* medicine physician), two *smen-pas* (traditional clinical assistant) and a Pharmacognosist of MSP (see Acknowledgement for their details) (total participant *N* = 8). These expert participants were selected using convenience and purposive sampling method. They were consulted mainly based on their vast experience in the areas of traditional clinical practices, university lectureship, herbal formulations, field identification and collection of medicinal plants. The consultation with these experts especially on the clinical, teaching and medicinal plants identification practices were carried out using one-one in-depth question—answer sessions either through Facebook chat or an email correspondence. The topics of open discussions varied depending upon our understanding of the BSM practices.

The systematic process in documenting observations and learning from the focus group discussion was performed as previously described by Wangchuk et al. [8, 9]. Both the qualitative and quantitative information was gathered and retained in our research notes, Facebook chat account and the email correspondences. *Drungtshos* (*N* = 5) (see details in the Acknowledgement) were involved to triangulate the data sources in this paper by engaging them to proofread the manuscript, authenticate the information and let them raise their comments, which we have incorporated in the paper.

### Research team and reflexivity

While the first author obtained his M.Sc. and PhD credential in medicinal/natural products chemistry from the University of Wollongong in Australia, the second author obtained his higher national diploma quality assurance certification from Hull College, UK and postgraduate diploma in GMP from the Swinburne University of Technology in Australia. As a Senior Researcher and a Quality Manager at MSP for more than 13 years, we have worked closely with the Bhutanese *So-wa-rig-pa* communities - mostly traditional physicians at the FoTM, NTMH and MSP through which the cumulative information was gathered, processed and reported here. During that timeframe we have observed, recorded, participated in many traditional *So-wa-rig-pa* meetings, conferences and group discussions, involved in translation of *So-wa-rig-pa* medical uses and terms, provided trainings to the traditional physicians and clinical assistants on modern research methodology, investigated many research problems and even helped them devise quality assurance parameters to strengthen *So-wa-rig-pa* medical system in Bhutan. More specifically, both the qualitative and quantitative information was gathered using the method as described in Wangchuk et al. [8, 9] and also through Facebook based-chat interviews and the email correspondences (conducted as the part of first author’s PhD work in between 2009 and 2014). The study participants were our office colleagues working at the FoTM, NTMH and MSP as the *So-wa-rig-pa* practitioners, lecturers, administrator, researchers and the quality specialist. We have avoided biasness in our reasoning and no assumptions were made to enhance the current practices of Bhutanese *So-wa-rig-pa* medicine. The study is much of a factual description based on our ethnographic and content analysis, which is presented as-it-is practiced in Bhutan.

### Data analysis and reporting

Ethnographic data were recorded and were authenticated with the participants from FoTM, NTMH and MSP. The content analysis was used for synthesizing the data of oral accounts and the literature statements on the quality policy, regulation, safety and efficacy measures. The information was then compiled and represented into diagrammatic quality pillars for each organizations as: a) quality practices of university-based traditional medical education (FoTM), b) quality practices of hospital-based health care services (NTMH), and c) the quality practices of production-based medicines at MSP. Our first-hand experience as the researchers with the BSM for more than 13 years enhanced the data analysis, synthesis and discussions of this paper.

### Study limitations

This study did not cover the patients’ perspectives or that of policy and decision makers at the Ministry of Health in Bhutan. It is purely an observational and content analysis study and is designed to present the quality assurance system that is in practice at FoTM, DTMS and MSP at the time of study. A separate cross-sectional study is necessary to understand the patients' or general public’s perspectives on the quality of BSM.

## Results and discussions

The series of information obtained through appropriate ethnographic observation, content analysis, open forum discussions, social media interviews (Facebook and emails) and proofreading process were analysed, triangulated and presented here mostly in a descriptive manner under five different categorical sub-titles. They are: a) Policy frameworks supporting the Bhutanese *So-wa-rig-pa* health care system, b) Quality assurance system and practices of the Bhutanese *So-wa-rig-pa* medicine, c) Hospitals/health centers-based quality assurance of BSM health care services, d) University-based quality assurance of BSM education and human resource development, and e) Pharmaceutical industry-based quality assurance of medicine productions. Wherever relevant, the important policies and information has been quoted to stress the importance given by the government in Bhutan. Also relevant quality assurance documents from the WHO has been used as the standards to compare the quality parameters set by MSP during the manufacturing of traditional *So-wa-rig-pa* medicines. The triangulation of these data further helped us generate diagrams to illustrate our observations and also to formulate constraints, challenges and suggestions, which we described in details that follow.

### Policy frameworks supporting the Bhutanese *So-wa-rig-pa* health care system

The content analysis revealed that even before the WHO policy of integration was formulated, the third King of Bhutan, Jigme Dorji Wangchuk integrated the BSM with allopathic medicine as early as 1967. During our 13 years of observations, we saw the BSM being transformed into a sophisticated organization. For examples, the small training institute was being upgraded to FoTM under Khesar Gyalpo University of Medical Sciences, the NTMH was upgraded to the Department of Traditional Medicine, and the small Research and Quality Control Laboratory (RQCL) was upgraded to semi-mechanized MSP. Today, there are 58 traditional medical hospitals and units, which are established next to each modern hospital building, follows the mainstream medicine acts and regulations, and adheres to integrated policy of quality assurance system. The BSM has become one of the important cultural and traditional heritages of Bhutan and there are proper inspection and enforcement agencies to check if the quality of BSM is practiced at the acceptable level within Bhutan. This medical system is enshrined in the constitution [10] and it is stated that ‘The State shall endeavor to preserve, protect and promote the cultural heritage of the country’ and ‘shall provide free access to basic public health services in both modern and traditional medicines’. Bhutan 2020: A Vision for Peace, Prosperity and Happiness [11] states that, ‘We must continue to provide a place for traditional medicine in our system of health care and must seek to achieve further improvements in its quality. As these qualities become substantiated by scientific research, there is a growing need to integrate traditional medicine more effectively with the modern health care system’. The national health policy of the Ministry of Health [12] states that, ‘Focused efforts shall be directed towards making the BSM, the center of excellence in providing quality traditional medical services including wellness center that is recognizable at an international level’. The National Drug Policy [13], states that ‘The government shall promote and support research and local manufacture of pharmaceuticals including traditional medicines and that the Drug Regulatory Authority (DRA) shall be the agency that develops and implements most of the legislation and regulations related to quality, safety and efficacy of drugs and the accuracy of product information on pharmaceuticals’. The Medicines Act of the Kingdom of Bhutan [14] and Bhutan Medicine Rules and Regulation [15] mentions that ‘The pharmaceutical factories including the MSP should conform to the current Pharmaceutical Inspection Convention/Co-operation Scheme (PIC/S) Guide to Good Manufacturing Practice (GMP) for medicinal products, shall have a separate quality control unit with qualified staff and appropriate equipment to carry out quality tests of raw materials and the finished products, and shall register the medicinal products with the DRA of Bhutan prior to making commercially available in the market’. The Quality Assurance and Standardization Division (QASD) [16] states that ‘QASD shall support the process of continuous quality improvement through policy development and adoption, establishment of quality standards, training and implementation of quality strategies at health facility and health program levels.’ Bhutan Medical and Health Council (BMHC) mandate states that ‘The medical and health professionals should be all registered to promote competency and ensure the safety of health of the public’.

While the DRA and the BMHC are the independent external regulatory bodies that frame overall laws and regulate the quality of the medicines and services, the QASD is an internal body under the Ministry of Health which monitors and assess the quality of services and prepares the organizations for compliance to external regulatory requirements. Most of these policy frameworks, regulatory statements and the quality parameters are found accommodative of BSM concepts and requirements. This is a strong indication of the government support on the preservation, growth and promotion of BSM as a health care provider. The BSM, which is based on Buddhist philosophy, has strong quality and moral ethics, health concepts and manufacturing process, which can enrich the western-borrowed health quality concepts and parameters.

### Quality assurance system and practices of the Bhutanese *So-wa-rig-pa* medicine

We have observed that the BSM has made decent progress in all areas of infrastructure, human resources and quality assurance system ever since its integration with allopathic/modern medicine in 1967. Traditionally, according to *Drungtsho* participants, the quality of BSM is defined by *smen-pai-ju-druug* (Physician’s six merit/criteria) and *che-pai-yen-lag-b.duen* (translated as ‘seven quality attributes of medicinal procedures’ or ‘seven affectionate branches of medical practices’). In essence, the quality of BSM is determined by how the diseases are diagnosed appropriately through compassionate patient-centered approach by the physicians and facilitates development of the patient’s balanced health - physically, mentally and spiritually. According to WHO [17], the quality of a health system is defined as a process of seeking continuous improvements in the dimensions of acceptability, accessibility, equitability, efficiency, effectiveness, and safety. This study reveals that, the MoH have continuously strived to improve these six dimensions of quality in BSM through three main administrative and functional strategies: a) imparting quality BSM education and developing technically qualified and skilled practitioners at FoTM, b) establishing BSM hospitals alongside all modern hospitals and BHUs and providing free health care services throughout the country, and c) manufacturing quality medicines at MSP using pristine Himalayan medicinal resources.

### University-based quality assurance of BSM education and human resource development

Most of the participants who contributed and authenticated the information in this study were the alumni of FoTM (previously known as NITM). The documents and the websites of FoTM highlighted the institute being a premier tertiary institute in the country (with more than 48 years of experience) in providing quality BSM education. Its vision and mission [18] states that: ‘The FoTM will strive to achieve excellence in the design, development and delivery of *So-wa-rig-pa* education programs through research and innovation, blending rich ancient wisdom and modern science to make the programs relevant for the current health needs of the people. It will incorporate the values and principles of Gross National Happiness (GNH) as foundation for all educational programs and will aspire to enhance their quality through realization of the core values including love and compassion to patients, research and development and integrated approach to health care’. The content analysis of literature related to FoTM revealed that the FoTM has come a long way in terms of infrastructure development and the way the education has been imparted to students. The first Indigenous Training Centre was established at Dechencholing, Thimphu where eight students underwent three years *smen-pa* (diploma) courses and in 1978, five years *Drungtsho* (Bachelor of Science in Traditional Medicine) course was instituted under the Ministry of Health (MoH). Through interactions with *Drungtshos* and other BSM colleagues, we found that the first batch of students were recruited from among the Buddhist monks, *Gomchens* (lay priest), sons or daughters or relatives of already practicing *Drungtshos*, and the Semtokha school graduates of Institute of Language and Cultural Studies (ILCS). Quality of education, knowledge and practice varied from a teacher to teacher (determined by a lineage based practices) and also among the students. We observed that students whose parents were themselves *So-wa-rig-pa* practitioners enjoyed best status and earned more respect. Since the lecturers who were first recruited to teach the students at the NITM (now called FoTM) had their training and education from different masters and traditional medical institutes in Bhutan and Tibet, inconsistencies and discrepancies–mainly in secret Tantra knowledge and identification of medicinal plants–were more prevalent until mid-1980s. These problems appeared to have resolved by 1988 through the standardization of BSM knowledge, curriculum, pedagogy and introduction of botanical identification system. We have triangulated the data sources and standardized both traditional and botanical nomenclature of medicinal plants nomenclature as described by Wangchuk et al. [9]. The curriculum and duration for the B.Sc. in TM course was set for five years with additional six months of internship to maintain equivalence to the allopathic system of Bachelor of Medicine and Bachelor of Surgery (MBBS) courses.

**Fig. 1:**
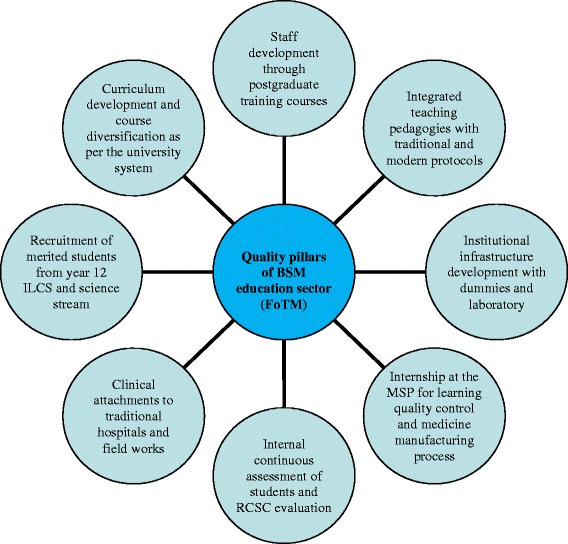
Eight pillars or strategies of quality assurance system practiced at FoTM in providing BSM education and training

Historical accounts have it that, in an effort to modernize and transform *So-wa-rig-pa* medicine into a scientifically-validated evidence-based medicine, the MoH recruited Dr. Paolo Morisco (Western Medical Doctor) to establish the Research and Quality Control Laboratory (RQCL) in 1990 with the help of an Italian Disarmo Sviluppo (DISVI) project. Through this project and under his technical expertise, Research Assistants were trained for three years at the NITM. To train the local students and scientifically validate the BSM, he further recruited other technical experts with multi-disciplinary qualifications in the areas of ethnobotany, pharmacy, pharmacognosy, chemistry, pharmacology and herbalism. The quality of education provided by the NITM was strengthened when it became a part of the Royal University of Bhutan (RUB) in 2003. Various changes, including the curriculum, were implemented in accordance to the requirements of the RUB system and guidelines [19]. The establishment of the ‘Traditional Medicine Research and Development Committee of Bhutan’ (TMRDCB) and the introduction of the ‘*Menjong Sorig* Journal’ (MSJ) in 2008 improved the quality of research that were carried out at NITM and the quality of research papers that were published in the journal. The first author of this paper (Phurpa Wangchuk), Drungtsho Tempa Gyeltehen and Dorji Wangchuk (NITM director) initiated the development of TMRDCB and MSJ and coordinated the research meetings and the publications of journal issues on an annual basis. The NITM and the Royal Institute of Health Sciences (RIHS) were formally transferred to the newly established University of Medical Sciences of Bhutan (UMSB) in 2013, which was in 2015 renamed as the Khesar Gyalpo University of Medical Sciences of Bhutan (KGUMSB). Today, it functions as the Faculty of Traditional Medicine (FoTM). A participant from FoTM stated that, ‘the FoTM now has separate newly constructed state-of-the-art administrative and academic block with built-in library facilities; a computer laboratory with Wi-Fi internet connections; modern lecture rooms equipped with liquid-crystal display projectors; a demonstration laboratory with facilities and dummies to learn human anatomy; and the traditional equipment for medicinal preparations. It follows the medical university policies, directives, education guidelines and the curriculum, which is revised every five years’. Our analysis of the FoTM textbooks, documents and curriculum revealed that there are about 28 traditional textbooks, which are used as the key teaching and learning resources – the main being *Gyud-zhi* (Four Medical Tantras) and *Shel-gong-Shel-phreng* (The Crystal Mirror and Rosary). The FoTM lecturer have adopted more progressive educational pedagogical methods including question-answer sessions, presentations, project-based learning, demonstrations, clinical assessments, spot identification, interacting with dummies, and field work training. The quality of the academic sessions also improved with the introduction of modern learning modules in addition to the traditional classical *So-wa-rig-pa* modules. The five-year academic program for *Drungtsho* course has a total of 49 modules with 660 credit hours out of which 39 modules are on *So-wa-rig-pa* medicine [20] and the remaining ten modules include research methodology, English language, information and communication technology, universal human values and professional ethics, analytical skill, modern anatomy and physiology, hospital management and sanitation, and the national health care system. The three-year *smen-pa* course has a total of 28 modules and 360 credit hours [21] with 21 core modules on general *So-wa-rig-pa*. ‘The Medical and Health Council Act’ [22], ‘Royal Government of Bhutan Medical and Health Council’ [23], and the ‘Disciplinary Proceedings for Medical Malpractice and Negligence Regulations’ [24] made it mandatory for the passing out graduates from FoTM to register themselves and obtain clearance for clinical practices and refrain from malpractices that would risk the safety of patients. The current FoTM practices, although not stated in the RUB norms, falls under the eight pillars/categories of quality assurance strategies as shown in Fig. 1.

These sets of FoTM’s practices resulted in producing high-quality graduates that have even surpassed other technical graduates from other modern technical universities both within the country as well as from abroad. These changes had ripple effects in quality of BSM education and students. For examples, in between 2007 and 2013, two FoTM graduates topped the Royal Civil Service (RCSC) examination [25] and all the graduates secured government job which were indicative of the quality of BSM education policy and courses offered by the institute. Getting selected in this RCSC examination is considered the stepping-stone to higher echelon of professional and leadership career in the country.

### Hospitals/health centers-based quality assurance of BSM health care services

**Fig. 2:**
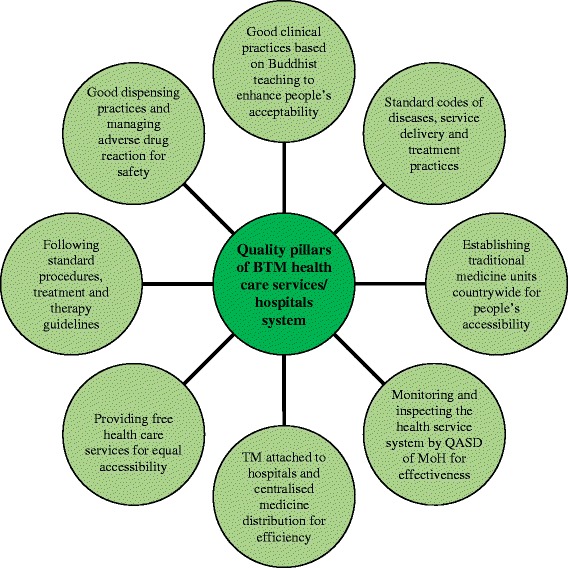
Eight pillars of hospital-based quality practices of BSM in Bhutan

To the best of our knowledge, there exist no specific policy or NTMH statement that mentions the need to adhere to the WHO definition of quality while providing traditional health care services. However, we have observed that the DTMS under the MoH have strived hard to address and attain many dimensions of quality, which we have depicted in Fig. 2 as the eight dimensions of quality pillars that are in a way related to the WHO definition of the quality of a health system. The WHO dimensions of quality of health care system includes achieving acceptability, accessibility, equitability, efficiency, effectiveness, and safety. Since BSM is closely knitted with Buddhism that is central to people’s beliefs, cultures and tradition, it has gained widespread acceptance from the king, government and the people. The Buddhist-based ethical principles and modern clinical norms practiced by the *Drungtshos* have also helped gain respect for the medical system. A study conducted in Thimphu by Lhamo and Nebel [26] showed that the attitude of people towards BSM was still good and encouraging, and that the treatment is sought by all ages, young and old and also across different levels of education. The practitioners themselves believe that BSM is a patient-centred health care delivery system where all patients are provided free access to it ever since its integration with modern health care system. Having the BSM alongside modern hospitals not only provided health care choices and access from the same building to the people but also helped preserve one of the country’s rich cultural heritages.

*So-wa-rig-pa* medicine dispensary was established at Dechencholing in 1968 and since then the government has expanded the services to all parts of the country with the objectives of making it more accessible. Currently, we found that there are 58 BSM centers/units attached to modern hospitals and Basic Health Units (BHU), which are distributed equitability in all parts of the country. The health care integration policy enabled the BSM to share the modern hospital building and other health resources that expedited the establishment of the traditional treatment centers in a cost effective manner. Accommodating both modern and BSM in the same hospital have maximized resource utilization and facilitated efficient cross referral of patients between the two medical systems. *Drungtshos* and *smen-pas*, with whom the authors have consulted and worked with for more than 13 years, said that modern doctors often refer to them the patients with chronic diseases such as sinusitis, arthritis, rheumatism, digestive and nervous disorder. On the other hand, the *Drungtshos* refer the patients that require surgeries and antibiotic treatment to modern doctors. The BSM has niche health care services including preventative care that focuses on lifestyle or behavioral adaptations. Lifestyle changes involve personal adaptations to the ever changing seasons, climates, environment, diets and other societal norms. The information on lifestyle changes also forms part of the daily prescription advice and counseling services provided by the physicians. Mental health and general wellness activities have already been explored by the DTMS, MoH in Bhutan. The BSM treatments have improved the health outcomes for many individuals and communities. However, the treatments provided by BSM practitioners varied among the individual *Drungtshos* and *smen-pas* depending upon their knowledge and experience.

*So-wa-rig-pa* medicines are generally considered safe, but like modern drugs it can result in overdose and severe side effects if patients fail to adhere to the prescription advice and counseling provided by the physician. Overdose and poisoning is also more likely to occur when the physicians or clinical assistants lack clinical experience. To detect, report and manage these adverse drug reactions and side effects, a Pharmacovigilance Center or the Adverse Drug Reaction Surveillance and Reporting System (ADRSRS) was established at MSP in 2007 (later transferred to NTMH). It is also a norm to report such incidents directly to the Post Marketing Surveillance Division (PMSD) and the Drug Regulatory Authority of Bhutan (DRAB). In addition, we found that numerous standard guidelines and reference materials were developed by NTMH through series of consultative workshops to provide BSM treatments with same quality, safety and effectiveness. Our analysis of the existing documents, guidelines and reference materials of the NTMH revealed that seven of them were guidelines that help BSM practitioners in providing quality, safe and standard health care services. They are:

i. Traditional classification of diseases and related health problems-2005 [27]. About 79 diseases that are prevalent in Bhutan are described, coded and standardized by this guidebook and helps the BSM practitioners in correct diagnosis and proper treatments.
ii. Standard treatment guide for traditional medicine-2006 [28].
iii. National traditional medicine professional service standards-2007 [29].
iv. Guidelines for detecting, reporting and managing adverse drug reaction-2007 [30].
v. Therapy guidelines for traditional medicine practitioners-2008 [31].
vi. Standard operating procedure for traditional medicine services-2008 [32].
vii. Monographs on the use of traditional medicine in primary health care-2012 [33].

These guidelines form the part of the continuing medical education (CME). The Drungtshos and *smen-pas* are trained on how to follow, operate and use these guidelines while providing the traditional medical health care services.

### Pharmaceutical industry-based quality assurance of medicine production

Traditionally, the quality, safety, and effectiveness of any medicine/drug is believed to be determined by the quality of the raw materials and how they are handled through collection and production processes. Like modern medicine, the quality assurance of BSM production is very complex. What makes it even more complex is its multi-ingredient formulations. Some BSM has more than 100 ingredients with each ingredient possessing assembly of complex chemicals. Hence determining purity or impurity profiles for BSM formulations is harder to achieve. It requires cross-disciplinary approach and extensive collaborations with many stakeholders including: farmers, raw material suppliers, traders and businessmen, farmers, park managers, foresters, conservationist, environmentalist, ecologist, horticulturist, *Drungtshos*, *smen-pas,* chemist, pharmacognosist, pharmacist, pharmacologist, ethnobotanist, quality control manager, drug regulatory inspectors, planners and administrators. Besides, it requires careful approach to see that while modern scientific quality control protocols are introduced, the ancient wisdom of BSM is not lost or discarded. Therefore, it is apparent that huge financial resources is required to establish and run the mechanized BSM factory that is accommodative of both modern standards and empirical traditional practices.

From historical accounts of MSP, we found that between 1980s and 1990s, numerous international funding bodies including World Health Organization (WHO), Italian DISVI project, non-governmental organization and two successive European Union (EU) projects (Phase I in 1994–1998; Phase II in 2006–2009) were brought in to establish the MSP with modern infrastructure, mechanize the medicine production, strengthen human resource capacity, conceptualize corporatization of MSP and address the long term sustainable supplies of traditional medicines. Under these projects, various technical experts/assistants (TAs) from Europe, India and Thailand have been recruited in Bhutan and their technical inputs can be observed in the current MSP infrastructures, research activity settings, quality control system, Good Manufacturing Practices (GMP) and Good Laboratory Practices (GLP). While modern scientific approaches including the Good Manufacturing Practices (GMP), Good Laboratory Practices (GLP), Good Collection Practices (GCP), Good Dispensing Practices (GDP), and the Total Quality Control System (TQCS) were introduced, the ancient ethnoquality practices were retained, preserved or slightly adapted to meet the current needs.

**Fig. 3:**
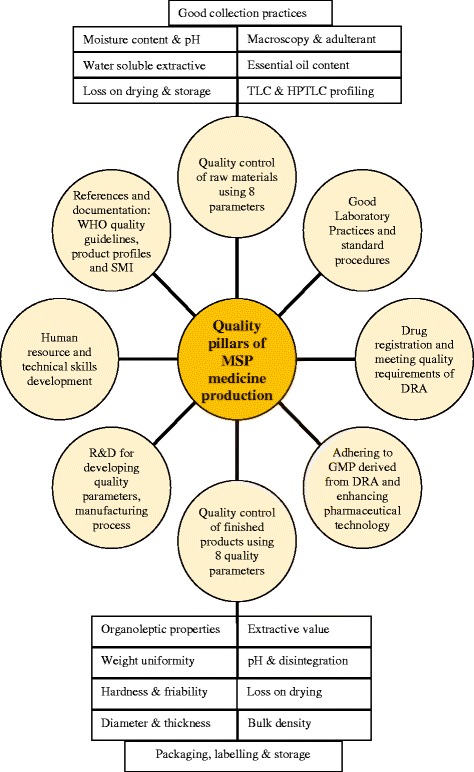
Eight pillars or strategies of quality assurance system practiced at MSP in manufacturing BSM

Based on the information gathered during this study and also observed by the authors while working at MSP in between 1999 and 2014, it is evident that the MSP have capitalized on improving the eight pillars of quality assurance norms or practices while manufacturing the traditional medicines (as shown in Fig. 3). Our assessment found that the quality of the manufactured medicines has been monitored in three main tiers at MSP. They are a) quality assurance in the field during the collection of medicinal plants, b) laboratory-based quality control testing of raw materials, pre-processed materials and manufactured herbal products, and c) quality control of labels and packaging materials. The raw materials include plants (as bulk ingredients), minerals, precious metals and animal parts.

#### Field-based quality control of the collection of medicinal plants

Collection of medicinal plants is the important activity of MSP. Correct identification of plants from the fields and the markets is considered the first crucial stage in assuring the quality, safety, and efficacy of medicines [9]. The field-based quality control protocol was reported previously [8]. In BSM, there exist an ancient written code of conduct or doctrine called *g.ches-pai-yan-lag-b.dun* (can be translated as ‘seven quality attributes of medicinal procedures’ or ‘seven affectionate branches of quality practices’), which ensure the quality of medicinal plants while collecting in the field. These seven quality doctrines include: a) correct identification of medicinal plants, b) collecting medicinal plants from the right natural habitat, c) following appropriate collection season and time, d) processing, drying, pre-processing and detoxifying the plants (wherever prescribed), e) using appropriate BSM recommended drying methods, f) proper storage and g) spiritual empowerment of herbs. The ethnoquality doctrine resembles ‘Good Collection Practices (GCP). During the collection season, the farmers’ responsibility is to collect the plant materials from the field based on the prior plant collection training they have received and the list provided to them by MSP. Other important post harvest care (as prescribed in the traditional medical texts), storage and spiritual empowerment are carried out by the MSP field-work team comprising pharmacy assistants, quality control staff and a *smen-pa*. Although detoxification of plants should be done during the fieldwork, they are currently carried out in the MSP because of lack of adequate facilities in the fields.

#### Laboratory-based quality control of raw materials

This field quality control system of the medicinal plants is supported by the laboratory-based scientific pharmacognostical and the phytochemical studies. In the context of quality-setting studies carried out at MSP, pharmacognostical studies mainly involve retention of herbarium specimen of each plant collected from the field for authentication, setting reproducible in house quality control standards and parameters for these medicinal plants, and developing plant monograph. Our literature search revealed that the WHO had released a series of plant monographs [6, 34–37] containing standardized methods, limits and other quality control parameters. However, our assessment of these documents showed that only few low altitude medicinal plants species have been covered by these WHO monographs and none of the 116 high altitude medicinal plants that are currently used in BSM were incorporated. Some of the quality parameters described in this monographs are beyond the affordability of many developing nations and are also very lengthy process, which is not feasible when hundreds of plants have to be screened for quality. As a result, an in-house quality control standards and parameters were developed by MSP, with the help of the chemist and pharmacognosist, for some high altitude medicinal plants (HAMP) following the formats and quality control protocols set by WHO [5, 38]. The MSP have later compiled two volumes of monographs on 40 HAMP [39, 40] and a Handbook on Quality Control of Raw Materials [41]. These monographs and quality control handbook are currently used as the standard references for the laboratory quality analysis of raw materials. The quality parameters included in those reference books includes the following: a) macroscopical examination and organoleptic characteristics, b) microscopical and cellular analysis (not practiced currently in routine QC at MSP), c) foreign matter, d) essential oil content, e) loss on drying, f) moisture content by azeotropic distillation, g) physio-chemical analysis, h) Thin layer chromatography (TLC) fingerprinting and i) pH. The physio-chemical analysis includes the determination of percentage values of: total ash, acid insoluble ash, water soluble ash, alcohol soluble and water soluble extractives. The TLC is a good quality profiling method as it portrays unique signature of individual plants for their identification but it is considered expensive for analyzing bulk samples.

**Fig. 4:**
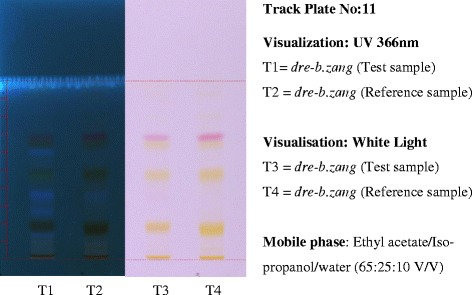
HPTLC profile plate developed by MSP quality control section for raw materials. T2 and T4 are the metabolite profile of reference samples from previous batch of raw materials (stored as reference drug). When the metabolite spots observed in the test sample conforms to that of the reference sample as observed in Track Plate No:11 (developed for *dre-b.zang* or Safflower in English), the test sample passes its identity test. The presence of adulterant should show different profile to the reference drug

Although more advanced equipment such as High Performance Liquid Chromatography (HPLC) and Ultraviolet (UV) spectrophotometer had been installed at MSP through European Union project assistance, they were found underutilised for monitoring the quality of medicinal plants and finished products due to high running cost and complex multiple ingredients especially in finished products. Recently, additional high-tech equipment called High Performance Thin Layer Chromatography (HPTLC) has been acquired and is under heavy utilization. This HPTLC is a powerful, fast and cheaper tool for metabolite fingerprinting (Fig. 4) and in analysing the quality of both plant and finished product samples. It is widely used across the globe in quality analysis of herbal traditional medicines and is the most appropriate and preferred analytical technology for complex mixtures of unknown therapeutic agents.

Although there is lack of research and development facilities at MSP to carry out advance molecular, phytochemical and pharmacological studies, the first author of this paper carried out few studies to scientifically validate the ethnopharmacological uses of BSM medicinal plants in collaboration with the University of Wollongong and James Cook University in Australia, and Srinakharonwirot University (SU) and BIOTEC in Thailand in between 2002–2004 and then in 2010–2014. These collaborative projects achieved three objectives: a) identified the marker chemical compounds of the plants which is used for developing monograph and quality control parameters b) validated the claims of the BSM uses of the plants providing scientific basis, and c) discovered new drug lead compounds. These scientific studies also revealed that out of 229 species of medicinal plants currently in use at MSP for manufacturing different polyingredient formulations, 55 species were endemic to the Himalayas and 33 of them were never studied before [42]. Out of 33 Himalayan species that were not studied, 10 species: *Aconitum orochryseum* [43, 44]*, A. laciniatum* [45]*, Ajania nubigena* [46], *Codonopsis bhutanica* [42], *Corydalis callaintha* [47], *C. crispa* [48], *C. dubia* [49], *Meconopsis simplicifolia* [50], *Pleurospermum amabile* [51, 52], *Ranunculus brotherusi* [53] and *Tribulus terristris* [53], were studied by the first author for their phytochemical and pharmacological activities employing the ethnodirected, biorational, and bioassay-guided strategies and protocols over a period of six years. These scientific data aided development of monograph and quality control parameters for the studied species of medicinal plants.

#### Laboratory and GMP-based quality control of manufactured products

Assessment of the formulary book [54] showed more than 100 polyingredient-finished product formulations are being described. However, only 94 of them are included in the current Essential Drug List (EDL) of Traditional Medicines that is being reviewed every after three years by the DTMS (MoH) based on the morbidity pattern and emerging need. The EDL committee members review the list of drugs that are in use in the hospitals and BHUs after collecting necessary feedback from the traditional clinicians across the country. Our analysis of the list of medicines that are manufactured at MSP showed that the main dosage forms includes pills (30.76 %), tablets (27.88 %), capsules (21.15 %), powders (10.57 %), ointments (4.80 %), syrups (1.92 %) and others (4.80 %).

Our observation was that MSP initially followed the European GMP model. Recently, the MSP has adopted the national GMP standard based on the Pharmaceuticals Inspections Cooperation Scheme (PIC/S). Traditionally, each polyingredient finished product contain mixture of different components in definitive ratios and therefore, the quality has to be evaluated with an understanding of the complexity of the raw materials used. *Drungtshos* and *smen-pas* believe that diseases are treated with complex mixtures of medicines since diseases are the expression of/disruption of the balance of five cosmo-physical elements (wind, fire, water, earth and space). Even the medicines are the expression of these five elements and the delicate balancing of these elements creates what is toxic, what is medicinal and which plants are of high grade or quality. A special feature of BSM medicinal products, as asserted by BSM practitioners, is that it is a multicomponent system containing: a) pharmacologically active substances, b) compounds which by themselves are not pharmacologically active but influence the biological effectiveness of active substances, c) neutral agents that serves as vehicle or acts as neutralizers of the toxic matters. Therefore, the advantage of using the multicomponent products is that the synergism among the active and non-active principles makes the medicine more powerful potions to treat complex diseases.

**Fig. 5:**
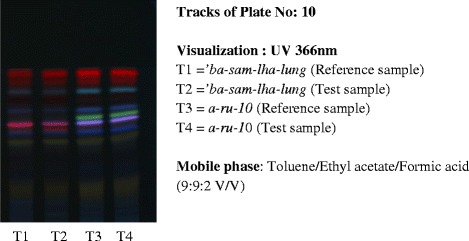
HPTLC profile of two finished products-*ba-sam-lha-lung* (T2) and *a-ru-10* (T4). T1 and T3 are the reference samples from previous batch that passed the quality and has been retained as standards. When the profile of the test sample is similar to that of reference sample in terms of number of bands, colour, size, and R_f_ values as in the Track Plate No:10 above, the test sample passes QC test for its identity test

Given the intricate BSM paradigm and multifaceted polyingredient formulations, strictly assessing their interactions, synergism and antagonism among constituent mixtures have been always challenging. Until now, the quality control of finished products depended heavily on assessing their organoleptic and physiochemical properties and very little was done to explain the mode and mechanism of action of BSM drugs. However, actions were being taken by MSP to lay down methods, standards and parameters for quality control of BSM manufactured products/drugs and even the manufactured product monograph has been developed. Quality standard of individual finished product is prescribed in this finished product monograph. Some general methods and reference points used in the finished products monograph are adopted from the British Pharmacopoeia [55] and the internally established standards. Once the final product, which is manufactured through series of steps in the production section, reaches the manufactured product quarantine, the quality control technicians test them for an overall quality following the prescribed quality control parameters in the product monograph. The quality parameters used for screening the quality of the manufactured products includes: a) description and organoleptic characteristics, b) hardness, c) friability, d) thickness, e) extractive value, f) uniformity of weight/dosage/weight per mL, g) pH value, h) disintegration, i) percentage loss on drying, j) bulk density, k) dosage forms and product identity, l) diameter m) thickness, and the o) HPTLC profile (Fig. 5). Quality control of label and packaging materials (includes dimensions, GSM, design, visual effect and defect and print quality) are also carried out to ensure their compatibility for packaging of manufactured product. The technical staffs responsible for the labeling and packaging section of MSP are required to sit for competency examination conducted by the DRAB. If the product fulfills the quality screening standards prescribed in the individual monograph, the products passes the quality test and are released to the Ministry of Health who distributes them to BSM hospitals and the BSM units across the country. However, if the final products don’t meet the quality requirements prescribed in the monograph, they are either sent back for re-processing or rejected. Rejection of a manufactured drug is very expensive when produced in bulk as one product contain many expensive ingredients and therefore, all preventative in-process controls during the production are mandatory.

The medicines that are manufactured at MSP for the first time and are used for treating the diseases must be registered with DRA. The DRA regulations including the ‘Bhutan medicine rules and regulations-2012’[15] and the ‘Guideline for application for registration of medicinal products-2013’ [56] states that all the finished products or BSM drugs must produce enough dossiers/documentations to prove their quality, safety and efficacy before releasing them into the hospitals and BHUs. Gathering the existing documents in this regards, we found that there exist heaps of products registration documents which include: a) company profile, b) manufacturing license, c) product profile, d) quality profile, and e) guidance of documentation system. The company profile documentation comprises an organization chart, vision and mission statement, quality policy of an organization, overall list of human resources, list of qualified technical persons, types of medicines produced, capacity of production, list of available pharmaceutical machineries, list of quality control instruments, and the list of key competent persons in the production and the quality control. The product profile for each medicine submitted for registration must have the information including: batch manufacturing formula, list of raw materials, product name, batch size, dosage form, pack size, manufacturing instruction, in-process quality control procedures, list of ingredients, a copy of original *So-wa-rig-pa* text references for each ingredient, a copy of original *So-wa-rig-pa* text references of the finished product formulary, manufacturing process flow chart, pre-processing method of raw materials, labelling and packaging instruction, and the price structure. The quality profile includes: certificates of analysis (CoA) of raw materials, CoA of manufactured products, CoA of labelling, CoA of packaging, stability study report (both accelerated and real time), adverse drug reaction reporting protocol, QC methods, QC release procedures and the stability protocols. These CoAs and protocols are to be appended with products specifications, inserts and product samples.

Documentation system consists of three components a) product profile which defines description, strength, reference method, packaging type and dosage form, b) quality profile defines manufacturing process, list of equipment both in production and quality control laboratory, content of active ingredients in the manufactured products, official methods used, report of raw materials and test methods, labels and packaging material, c) pharmacology profile is currently limited to the traditional uses only and supplying the BSM literatures as references or as supporting documents suffices the legal requirement for this profile. Although these regulatory requirements are mostly based on the western-borrowed pharmaceutical concepts that are very often difficult to fulfil, it improved the systematic documentation system of the BSM manufacturing process and enhanced the overall quality of the medicines.

### Constraints, challenges and suggestions

We have observed that the quality assurance system of BSM have drastically improved in comparison to the day when it was first integrated with the modern health care system in Bhutan in 1967. Quality assurances have become part of the good governance system and all three organisations (FoTM, DTMS and MSP) try to constantly adjust to the current needs of the society in the country. There are also many health and drug related regulations and enforcement agencies to inspect and monitor the ethical practices of the practitioners, hospitals and manufacturer. However, despite many improvements and system in place, there are various issues confronting the quality of BSM and the triangulation of data obtained from content analysis, observations and open group discussions confirmed this. The FoTM lacks qualified and experienced lecturer who could conduct independent inquiry-based research on BSM. Research is a building block of any university or institution and for FoTM, it is even more crucial to have research component built in the curriculum. Without research component, both the lecturers and the students would be learning only what is being written in the ancient textbooks. These written concepts need validation through research, which would lead to the development of new knowledge. The KGUMSB, with new leadership, is expected to remodel FoTM into a research-based institute.

The DTMS has established a research section but there is need for qualified staff to lead the section. There are 58 BSM centres/units in total and few of these units’ lack *Drungtshos*. To provide quality and safe services, qualified and competent professionals must run all these units in the country. Therefore, there is need for more *Drungtshos*. In addition, *Drungtshos* and *smen-pas* have raised the issues of the shortage of medicine supply in their units. The short supply of required medicine can affect the quality of traditional health care services. While many standard treatment guidelines and standard operating procedures (SOPs) have been endorsed and implemented, there is concern that few freshly appointed *Drungtshos* and *smen-pas* lack confidence to deliver the standard services. The training on using guidelines and SOPs are conducted as a part of in-service CME. Instead of having the course as a CME component, it would be worthwhile to put them as a part of FoTM curriculum.

MSP is the nerve-centre of BSM and any shortfalls in the medicine productions would immobilise the whole traditional medical system of a country. Sometime the product fails to meet the quality standard and re-processing the formulation prolongs the release of the manufactured products on time. The content analysis showed that Lingzhi (in the North) and Langthel (in the central parts of Bhutan) have been the collection centres for medicinal plants in Bhutan for more than 48 years. Triangulating the information collected, we realised that there is need to establish new collection centres across the country to meet the demand for medicinal plants. This would not only improve the sustainability of medicinal plants supply to MSP but doing this would also ease the pressure on medicinal plants growing in the existing collection centres. Other issues affecting BSM is using the ingredient substitutes to meet the production dateline. Although this practice is described in the ancient traditional medical textbooks, substituting the original ingredient with the other inferior ingredient is a scientific concern for quality, safety and efficacy. Some formulations contain as much as 35 different types of ingredients. This makes it complex and harder to assess the quality on a daily basis. The MSP have made good progress in the quality control of raw materials. However, there is need to improve the quality dimensions of manufactured products. There is urgent need to establish additional quality parameters to screen the manufactured products for heavy metals, pesticide residues, cytotoxicity, microbial limits, parasitic infestation, biological and chemical contamination. Clinical studies are recommendable for BSM to become an evidenced-based medicine.

## Conclusions

The integration of BSM with the modern health care system in 1967, under the command of the third king Jigme Dorji Wangchuk, was the foundation for quality assurance system in Bhutan. Having received the government’s support and wider people’s acceptance, there is no reason for the BSM not to flourish and improve its quality of health care services. Three functional organizations including FoTM, NTMH and MSP have established their image and brand as ‘*Man-jong So-wa-rig-pa’* (often spelt as ‘*smen-ljong-g.so-ba-rig-pa’*) and have made decent progress over the decades. *Drungtshos* and *smen-pas* receive free ancient medical education and knowledge delivered through integrated curriculum (traditional and modern pedagogies integrated). Unlike in the past, the traditional medical students are selected from the high achiever’s list of School of Language and Culture Study (ILCS) and most preferably with the science background. Continuous training for 5 years (*Drungtsho* course) and 3 years (*smen-pa* course) supported by clinical attachments to NTMH, internship at MSP, and the field works at the collection centers prepares them to take up the highly coveted job in the government run district hospitals and Basic Health Units in the country.

Based on the job vacancies, the *Drungtshos* and *smen-pas* are selected by the RCSC and are compulsorily registered with the BMHC (responsible for ensuring their ethical, professional and skills standards). The standard treatment guidelines, protocols and refresher course provided by NTMH guide them/BSM practitioners in providing quality and safe traditional health care services. Trained in health and sanitation, reporting adverse drug reactions, and good dispensing methods, the BSM practitioners are equipped to handle safety problems and work hand-in-hand with the modern doctors through cross-referral system in ensuring overall quality of the health services delivered through 58 TM centers.

The medicines that the BSM practitioners prescribe are manufactured at MSP using the country’s own organic medicinal plants that grows in the pristine Himalayan mountains. Monitoring the quality of medicinal plants at the sources and also in the laboratory using scientific quality control parameters ensure the total quality of the medicine manufactured. While ancient wisdom of BSM related to quality assurance system were retained, modern scientific studies were introduced, which made it unique by its own standing. Quality control system at MSP was inspired by the WHO guidelines and European GMP. The MSP have registered 75 polyingredient formulations with the DRA that regulates stringent (by local standard) manufacturing process and quality control measures through registration and regulatory audit in the country.

We argue that the quality control system in Bhutan manages to reach the high standards required by modern science while retaining and integrating traditional notions and measures of quality as enshrined by BSM. However, many efficacy and effectiveness stories remains anecdotal whose efficacies are based on the BSM textbooks or first-hand experience of *Drungtshos*. These claims need to be substantiated by the chemical and clinical studies, which is partially lacking at the MSP due to limited technical experts and financial resources. The safety and effectiveness of BSM can be substantiated only through clinical studies and such studies can be conducted at relatively low cost as studying traditional medicines does not require pre-clinical nor toxicology studies. Few studies carried out by the first author on 11 medicinal plants [57, 58] in collaborations with UOW and JCU in Australia, and SU and BIOTEC in Thailand, resulted in the: a) elucidation of five new phytochemicals, b) verification of the ethnopharmacological uses of these medicinal plants, and c) discovery of new antimalarial drug lead candidates. These scientific data form the basis for developing plant monograph and quality control parameters with the known marker compounds. Remaining plants can be investigated using similar strategy and protocols. It is proper to first focus on the scientific studies of single ingredient. The metabolomics involving mass Spectrometry (MS), Infrared Spectroscopy (IRS), Gas Chromatography Mass Spectrometry (GCMS) and Nuclear Magnetic Resonance (NMR) could help us understand the complex chemical mixture responsible for the efficaciousness of the BSM.

Immediate attention can be given to the following: a) focus on studying individual medicinal plants and creating metabolomics fingerprints of bioactive marker compounds including HPTLC profile which can be used for daily quality control evaluations, b) carry out advanced molecular and clinical studies on the finished products to substantiate the ethnopharmacological uses, c) check the chemical and bioactivity variations between the wild types and the cultivated species as chemical profile would be different depending upon their nutrient availability, which could affect the quality of medicines, and d) collaborate with other *So-wa-rig-pa*-practicing countries to identify existing differences and similarities in terms of education and clinical practices, plants uses and formulations, research and development activities, and the quality assurance of the raw materials and finished products.

The BSM including medicinal plants program in Bhutan is one of the sustainable vehicles of GNH. For patients ˗ its formulations are a solace of treatment and cure; for BTM and *So-wa-rig-pa* practitioners ˗ it is a source of employment; for pharmaceutical organizations ˗ it is a potential chemotherapeutic pool waiting for biodiscovery [59]; and for farmers ˗ it is a tool for income generation and poverty eradication [60]. Overall, it accelerates the health and well-being of the society and produces happy society. Failing to maintain the quality of BSM would negatively affect the sustainability of the health care system and the networks of medicinal plants stake holders including patients, farmers, brokers, business people and buyers, MSP, TMH, FoTM, MAPS and the NBC. Therefore, continuous efforts to improve the quality of BSM are essential and require immediate government investments in developing advance research and development facilities.

## Abbreviations

BHU, Basic Health Unit; BMHC, Bhutan Medical and Health Council; BSM, bhutanese *So-wa-rig-pa* medicine; BTM, Bhutanese Traditional Medicine; CAM, complementary and alternative medicine; CoA, certificate of analysis; DISVI, Italian Disarmo Sviluppo; DRAB, Drug Regulatory Authority of Bhutan; DTMS, Department of Traditional Medicine Services; EDL, essential drug list; EU, European Union; FoTM, faculty of traditional medicine; GCMS, gas chromatography mass spectrometry; GCP, good collection practices; GDP, good dispensing practices; GLP, good laboratory practices; GMP, good manufacturing practices; GNH, gross national happiness; HAMP, high altitude medicinal plant; HPLC, high performance liquid chromatography; HPTLC, high performance thin layer chromatography; ILCS, Institute of Language and Culture Studies; IR, infrared; JCU, James Cook University; KGUMSB, Khesar Gyalpo University of Medical Sciences of Bhutan; LAMP, low altitude medicinal plant; MAPS, medicinal and aromatic plants section; MBBS, bachelor of medicine and bachelor of surgery; MoH, Ministry of Health; MS, mass spectrometry; MSJ, manjong sorig journal; MSP, Menjong Sorig Pharmaceuticals; NBC, National Biodiversity Centre; NITM, National Institute of Traditional Medicine; NMR, nuclear magnetic resonance; NTMH, National Traditional Medicine Hospital; PIC, pharmaceutical inspection convention; PMSD, Post Marketing Surveillance Division; QASD, Quality Assurance and Standardization Division; QC, quality control; RCSC, Royal Civil Service Commission; RIHS, Royal Institute of Health Sciences; RQCL, research and quality control laboratory; SU, Srinakharinwirot University; TA, technical assistant; TLC, thin layer chromatography; TM, traditional medicine; TMH, Traditional Medicine Hospital; TMRDCB, Traditional Medicine Research and Development Centre of Bhutan; TQCS, total quality control system; UMSB, University of Medical Sciences of Bhutan; UOW, University of Wollongong; UV, ultraviolet; WHO, World Health Organization
